# Investigating the diagnostic accuracy of physical examination in digital nerve injury: a study within a trial

**DOI:** 10.1177/17531934251410456

**Published:** 2026-02-11

**Authors:** Ciarán O’Hanlon, Matthew D Gardiner, Loretta Davies, Dominic Power, Abhilash Jain, Jonathan A Cook, David Beard, Justin Conrad Rosen Wormald

**Affiliations:** 1 Nuffield Department of Surgical Sciences, University of Oxford, UK; 2 Surgical Interventional Trials Unit, Nuffield Department of Orthopaedics, Rheumatology and Musculoskeletal Sciences, University of Oxford, UK; 3 Department of Hand and Peripheral Nerve Surgery, University of Birmingham, UK; 4 Oxford Clinical Trials Research Unit, Nuffield Department of Orthopaedics, Rheumatology and Musculoskeletal Sciences, University of Oxford, UK; 5 NHMRC Clinical Trials Centre, Faculty of Medicine & Health, University of Sydney, Australia

**Keywords:** hand injuries, peripheral nerve injuries, physical examination, clinical trial, diagnostic accuracy, study within a trial

## Abstract

**Introduction:**

Digital nerve injuries are the most common peripheral nerve injuries, usually diagnosed through physical examination and confirmed by surgical exploration. Exploration, while important for informing management, carries risk and associated healthcare costs. The diagnostic accuracy of physical examination in identifying complete nerve lacerations requiring repair remains uncertain. This study within a trial (SWAT) aimed to determine the positive predictive value (PPV) of specialist physical examination in identifying complete digital nerve lacerations requiring repair.

**Methods:**

This prospective SWAT was embedded within the NEON (Digital Nerve, Suture or Not) randomized controlled trial (ISRCTN16211574). Adult patients presenting with suspected complete digital nerve injury in flexor zone 2 across nine UK NHS hospitals (September 2020 to March 2024) were included. Physical examination was performed by hand surgery specialists, with surgical exploration as the reference standard. The primary outcome was the positive predictive value (PPV) of physical examination for complete laceration requiring repair.

**Results:**

Of 593 referred patients, 242 underwent surgical exploration. Complete laceration was confirmed in 137 (true positives), while 105 had partial or no visible lacerations (false positives), yielding a PPV of 57%.

**Discussion:**

Specialist physical examination was unable to reliably predict complete digital nerve injury, resulting in a high false positive rate, with 43% of explored patients not requiring repair. Despite this limitation, surgical exploration remains pivotal, as no alternative bedside test currently provides sufficient accuracy. These findings highlight the urgent need for improved point-of-care diagnostic modalities to support surgical decision making and informed patient consent.

**Level of evidence:**

II

## Introduction

Digital nerves are the most frequently injured peripheral nerves with an estimated incidence of 8.4 per 100,000 in a typical northern European urban setting (Evertsson et al., 2023). Digital nerve lacerations often result from sharp trauma and are identified via physical examination. The extent of the laceration is then confirmed through surgical exploration. Standard practice prioritizes early diagnosis and microsurgical repair of complete lacerations under loupe or operating microscope magnification (British Society of Surgery of the Hand Standards of Care in Hand Trauma: Nerve Trauma Management, 2024).

However, surgical exploration itself carries a small risk of iatrogenic harm and has associated costs to healthcare systems. It is important that patients do not undergo exploration unnecessarily and are informed that even with highly suggestive clinical findings, digital nerves may not be injured to an extent warranting suture repair.

The diagnostic accuracy of physical examination in predicting severity and thus the need for digital nerve repair is unknown. Small retrospective studies estimate a positive predictive value (PPV) of 61–82% for physical examination performed by hand surgery specialists (Nassab et al., 2007; Rubin et al., 2020).

Therefore, this prospective study within a trial (SWAT), embedded within the NEON (Digital Nerve, Suture or Not) randomized controlled trial, aimed to further investigate the diagnostic accuracy of physical examination in identifying complete digital nerve injuries requiring surgical intervention.

## Methods

This study follows STARD 2015 reporting guidelines for diagnostic accuracy studies and Trial Forge reporting guidelines for randomized SWATs (Arundel et al., 2024; Cohen et al., 2016).

The NEON trial (ISRCTN16211574), is a two-arm, parallel-group, double-blind, multicentre RCT. Patients with suspected unilateral digital nerve injury underwent surgical exploration, and those with complete digital nerve injuries were randomized to microsurgical suture repair or nerve alignment alone.

### Inclusion criteria

This SWAT includes participants who were assessed in specialist hand clinics and underwent surgical exploration for suspected complete digital nerve injuries before randomization in NEON. Injuries were restricted to flexor zone 2 (from DIPJ to distal palmar crease) in any single digit, including the thumb and little finger.

### Exclusion criteria

Patients were excluded if they had bilateral injuries (both radial and ulnar nerves injured), lacerations outside flexor zone 2, closed injuries, infected wounds and non-isolated or multi-level injury (meaning common digital/wrist nerve injury or fracture). Patients were also excluded if they were unable to consent or comply with study follow-up procedures or if surgery took place later than 10 days after injury.

Recruitment took place across nine NHS hospital sites between September 2020 and March 2024. Consecutive patients with suspected digital nerves injuries identified in A&E or minor injuries unit were referred to hand surgery teams.

The primary test was physical examination performed by trainee or consultant surgeons in plastic and hand surgery units. The reference standard was surgical exploration under anaesthesia, identifying injured and uninjured structures within the zone of injury. A positive test was defined as a complete laceration; a negative test included nerves found completely intact or with incomplete transection not meeting the threshold for repair. True positives (TP) and false positives (FP) were recorded to calculate the PPV:

$$\mathrm{PPV}=\frac{\mathrm{TP}}{\mathrm{TP}+\mathrm{FP}}$$

True negative and false negative results were not obtained, as only patients with suspected complete digital nerve lacerations were included. Thus, summary measures such as sensitivity, specificity, or negative predictive value could not be calculated. The SWAT sample size was dependent on the NEON trial; therefore, no formal sample size calculation was performed. Analysis was based on observed data without imputation, with variables presented as frequencies and percentages, stratified by injury class.

## Results

**Figure 1.**
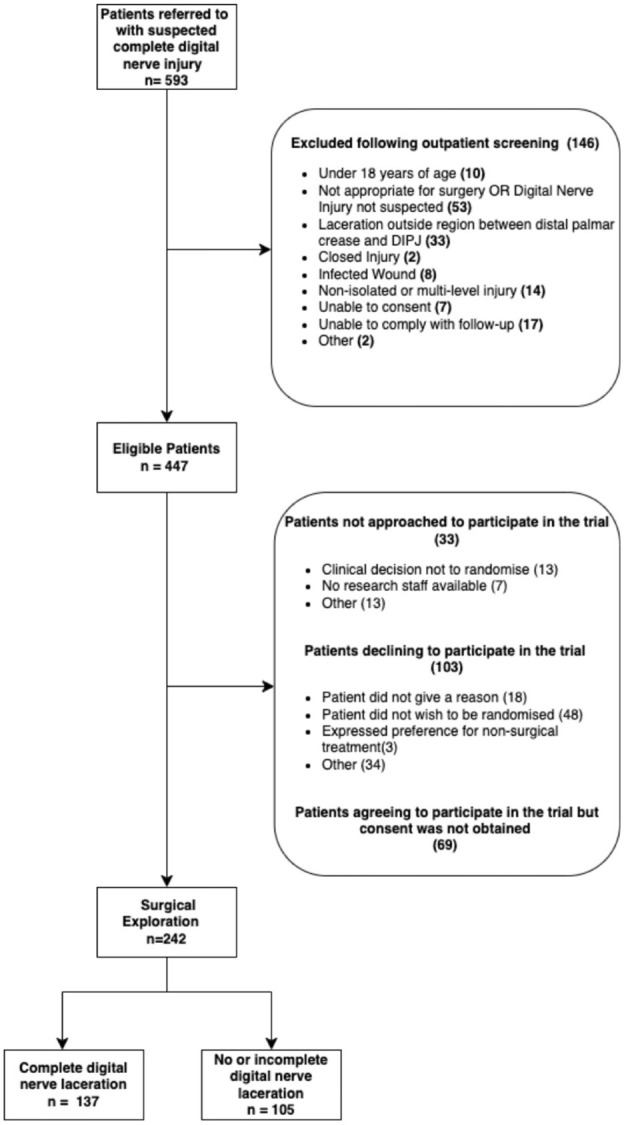
STARD 2015 flow diagram detailing reasons for exclusion of ineligible patients and reasons that eligible patients did not undergo surgical exploration.

A total of 593 patients were referred to the plastic hand surgery clinic with suspected complete digital nerve injuries, and 242 patients underwent surgical exploration (Figure 1). Of these, 137 had a complete laceration requiring repair (TP), while 105 patients had either no visible or partial lacerations not requiring repair (FP), the latter based on surgeon judgement.

Physical examination by clinicians in a hand surgery clinic had a PPV of 57% (137/242). In patients with absent sensation at the time of referral, the PPV was 76% (19/25).

## Discussion

This study found that physical examination by hand surgery specialists, despite its significant impact on clinical decision-making, has limited accuracy, identifying patients requiring surgical repair correctly in 57% of cases.

These findings align with a retrospective case series of 70 patients reporting a PPV of 66–82% for six clinical tests in diagnosing digital nerve lacerations (Rubin et al., 2020). In this study, isolated testing of light touch sensation had the lowest PPV (66%) compared with tests such as Dynamic-2-Point Discrimination (78%) and the wrinkle test (82%). Combining multiple testing modalities was found to further increase diagnostic accuracy; however, specificity was generally poor (28-62.5%). The authors did not distinguish between partial injuries and complete injuries requiring repair, which may explain the higher values observed.

Similarly, a retrospective case series of 27 patients with digital nerve lacerations observed a PPV of 61% for physical examination performed by hand surgery specialists in detecting injuries involving >50% of the nerve diameter in the hand overall (62% in Zone 2 specifically).

This SWAT demonstrated a high false-positive rate with 43% of surgical explorations performed after specialist assessment revealing intact nerves or partial lacerations not meeting the threshold for repair. Although these subgroups could not be separated, in the absence of concomitant injury, any exploration not leading to repair is potentially avoidable. This has implications for patient safety and resource utilization. While exploration under anaesthesia for suspected complete nerve injury is certainly clinically and medicolegally defensible, these findings support the need for more reliable point-of-care diagnostics to distinguish patients who require operative repair from those suitable for expectant management.

It is suggested that multi-modal assessment including clinical assessment, electrodiagnostic studies and neuromuscular imaging may improve patient selection for surgery following traumatic peripheral nerve injury (Barnes et al., 2022).

Electrodiagnostic studies such as nerve conduction studies or needle electromyography can facilitate localization and grading of nerve injuries in both traumatic and non-traumatic settings. However, sensory nerve action potentials cannot reliably distinguish neuropraxia from axonotmesis until Wallerian degeneration has occurred (typically 10–14 days post-injury), limiting utility in the acute phase of digital nerve injuries (Bourke et al., 2024; Ferrante, 2018).

In contrast, both MRI and ultrasonography can identify early post-traumatic findings, including nerve discontinuity, oedema and early muscle denervation, with sensitivity for some features improving over time (Graesser et al., 2025).

The diagnostic accuracy of magnetic resonance imaging (MRI) in detecting acute traumatic peripheral nerve injuries varies by injury type, nerve location and MRI protocol, but consistently demonstrates high sensitivity (75–93%) and moderate-high specificity (60–83%) in upper extremity nerve injuries (Foesleitner et al., 2025; Wade et al., 2019; Zhang et al., 2018). Evidence relating specifically to digital nerves is lacking; however, anticipated limitations include relatively high cost and a lack of availability (Bourke et al., 2024).

Ultrasonography has a limited penetration depth, which makes assessment of deeper structures challenging, but lends itself well to the assessment of digital nerve injuries. In the past, limited resolution of widely available ultrasonographic systems has limited diagnostic capabilities to the assessment of gross nerve integrity. However, with advances in high resolution ultrasound, 3D-tomographic software and the advent of high frequency transducers, nerves can now be visualized at the fascicular level (Murphy et al., 2023; Pušnik et al., 2025), which may signal a plausible shift towards the use of ultrasonography as a preliminary screening test in traumatic peripheral nerve injuries (Graesser et al., 2025).

Small prospective studies have evaluated the use of high-resolution ultrasound in diagnosing traumatic digital nerve injuries using surgical exploration as the reference standard. Umans et al. included 10 adults with 20 clinically suspected digital nerve injuries. Ultrasonography correctly differentiated between intact nerves and those requiring repair, as indicated by intra-operative findings. Of the five adults with sensory deficits and intact nerves, four required operations for concomitant tendon injuries; however, one patient could have avoided surgery for the sole purpose of digital nerve exploration (Umans et al., 2012).

Endo et al. reported on the diagnostic accuracy of long-axis ultrasound assessment in detecting traumatic digital nerve injuries and highlighted a potential limitation of ultrasound in the assessment of digital nerve injuries. In 15 surgically confirmed injuries, the laceration site could not be localized on long-axis imaging in approximately one-third of cases (4/15). Among visible injuries, expert readers correctly classified most complete lacerations (7/8) but fewer incomplete ones (1/3) (Endo et al., 2021). Given the small sample sizes in both studies, reported measures of diagnostic accuracy are interpreted cautiously.

Other immediate limitations of ultrasonography are that image acquisition quality and interpretation is considerably operator dependent (Graesser et al., 2025). Like MRI, availability is also a constraint, primarily driven by a lack of dedicated operators and poor reimbursement for scanning by non-radiologists. As the technology improves and interest in ultrasonographic peripheral nerve imaging grows, an emphasis on cross-disciplinary training and collaboration, alongside appropriate alignment reimbursement mechanisms, may help address these barriers (Bourke et al., 2024).

This study has some limitations. As discussed in the host trial, approximately 46% of eligible patients were not included, potentially impacting the representativeness of the study sample (Wormald et al., 2025). Additionally, the false-positive group could not be further stratified into partial vs. non-visible lacerations, which limits clinical interpretation of avoidable vs. justified surgical explorations. The study would also have benefited from more detailed characterization of assessor seniority and examination techniques to explore the impact on diagnostic accuracy.

However, internal and external validity are strengthened by the prospective, multi-centre design, which increases representativeness of both assessors and patients, and supports generalizability to outpatient hand and plastic injury clinics.

In conclusion, physical examination by hand surgery specialists cannot reliably identify digital nerve injuries requiring surgical repair, reinforcing the need for improved and more accessible diagnostic tools for traumatic digital nerve injuries. By quantifying diagnostic uncertainty, this study can support clinicians to obtain informed consent and manage patient expectations when discussing surgical interventions. The findings also have important implications for planning future trials in digital nerve injury, with fewer than anticipated patients being potential candidates for surgical intervention.
